# Effects of Extraction Methods on Physicochemical, Functional and Nutritional Properties of Protein Extracted from Mustard (*Brassica juncea*) Seed Meal Residue

**DOI:** 10.3390/foods15183294

**Published:** 2026-09-17

**Authors:** Piyathida Chanaim, Siraphat Taesuwan, Suphat Phongthai, Dayong Zhou, Qi Zhao, Kanyasiri Rakariyatham

**Affiliations:** 1 Faculty of Agro-Industry, Chiang Mai University, Chiang Mai 50100, Thailand; piyathida_cha@cmu.ac.th (P.C.); suphat.phongthai@cmu.ac.th (S.P.); 2 National Engineering Research Center of Seafood, School of Food Science and Technology, Dalian Polytechnic University, Dalian 116034, China; zdyzf1@163.com (D.Z.); zhaoqi@dlpu.edu.cn (Q.Z.)

**Keywords:** *Brassica juncea*, mustard seed, by-product, solid waste, plant protein, sustainable food, sustainable agriculture

## Abstract

Agro-industrial by-product valorization promotes the circular bioeconomy, food security, and sustainability by recycling waste products into reusable resources. This study evaluated various protein extraction methods for processed mustard seed (*Brassica juncea*) meal, including conventional alkaline (CON), enzyme-pretreated alkaline (ENZ), ultrasound-assisted alkaline (US), and enzyme-pretreated and ultrasound-assisted alkaline (ENZ+US) extraction methods. The protein content extracted from the various treatments ranged from 70.67% to 82.31% (molecular weights: 10–75 kDa), with morphology shifting from rough to smooth surfaces in enzymatic treatments. Viscozyme L hydrolysis of cell-wall polysaccharides in ENZ and ENZ+US treatments facilitated co-extraction of non-protein bioactives as shown by enhanced antioxidant capacities through hydrogen/electron donation mechanisms, including superior Folin–Ciocalteu reducing capacity (7.20–7.95 mg GAE/g), radical scavenging (DPPH•: 8.75–9.33; ABTS•^+^: 42.97–56.86 mg TE/g), and ferric-reducing antioxidant power (FRAP: 13.59–14.49 mg TE/g). Conversely, the US treatment may generate reactive species that reduce antioxidant capacity while increasing visual lightness (L*) and preserving in vitro digestibility (66.19%) at a level comparable to that of CON (67.63%). Meanwhile, the CON treatment exhibited the highest protein solubility across all pH levels, driving a higher emulsifying activity index (42.15 m^2^/g). In summary, this research demonstrates that *B. juncea* seed waste can be transformed into high-value functional ingredients by tailoring the extraction method to targeted product properties. However, low extraction yields present a significant bottleneck, and therefore industrial and economic feasibility will depend on future yield optimization in order to drive sustainable plant-based protein development.

## 1. Introduction

*Brassica juncea*, commonly known as “brown mustard” or “oriental mustard”, is grown and traded worldwide for a variety of culinary uses. Edible oil is extracted from the seeds, the leaves are consumed as a vegetable, and the seeds are used as a spice in various pastes and powders. The proximate composition of *B. juncea* whole seeds consists of fixed oils (32–36%), protein (23–26%), and crude fiber (6–7%), along with minor constituents, like moisture and ash [1]. In terms of composition, the seed meal or press cake generated via oil extraction comprises more than 50% of the initial seed mass and remains a valuable source of protein containing all essential amino acids [2,3,4,5]. Because it contains adequate levels of lysine alongside sulfur-containing amino acids, such as methionine and cysteine, which are typically limited in most cereal grains and oilseeds, respectively [1], mustard protein is highly attractive for plant-based dietary applications [6]. Consequently, it is utilized in aquaculture feed and animal nutrition, and has promising potential as a source for human food applications [7]. Several studies have demonstrated the nutritional fortification of baked goods, such as wheat bread, biscuits, and cookies, through the incorporation of mustard seed flour or seed meal [8,9,10]. Other studies have utilized mustard protein extracts not only for nutritional fortification, but also to improve the oxidative stability and physicochemical properties of foods and beverages, including beef patties, pasta, and fruit juices [11,12,13].

Regarding protein extraction methods, alkaline extraction followed by isoelectric precipitation remains the most conventional and widely adopted protocol for plant protein isolation, owing to its operational simplicity and cost-effectiveness. The process relies on the solubilization of proteins within an alkaline medium, followed by targeted sedimentation at their characteristic isoelectric point (pI). Although elevating the extraction pH can substantially enhance protein yields, the alkalinity must be carefully optimized to prevent severe, irreversible denaturation [14]. However, since plant proteins are often tightly trapped within cell walls and intracellular matrices, advanced green technologies are required to disrupt these networks and facilitate recovery. Specifically, enzyme-assisted extraction using carbohydrases breaks down plant cell wall polysaccharides, allowing efficient protein release without inducing structural denaturation. For instance, applications of cellulase and pectinase during aqueous extraction (enzymes’ optimal pHs with no alkalinity) enhanced the protein recovery yields from soy grit by approximately 10% [15]. Utilizing Viscozyme L, a multi-component enzyme containing a comprehensive array of carbohydrases (including arabanase, cellulase, hemicellulase, and xylanase), to assist alkaline extraction yielded a 56.27% protein extract, which was significantly higher than that obtained by conventional alkaline extraction alone [16]. Another non-thermal process, ultrasound-assisted extraction, can also increase extraction efficiency while reducing extraction time and solvent consumption. High-intensity ultrasound (20 kHz, horn-type)-assisted extraction significantly outperformed the conventional alkaline method for hempseed protein extraction by exploiting acoustic cavitation to achieve up to a 2.1-fold increase in yield, improved solubility and enhanced essential techno-functional properties, such as emulsification, foaming, and oil-holding capacity through structural particle size reduction [17]. Utilization of an ultrasonication bath (450 W) significantly outperformed the traditional water-bath method for rapeseed protein extraction, increasing extraction efficiency by over 35% at a low processing temperature of 35 °C, while yielding protein extract with emulsifying and foaming properties superior to those of soybean protein [18].

While protein extraction from *B. juncea* has been investigated, comparative studies regarding different forms of processed biomass, extraction methodologies, and their subsequent impacts on protein functional properties remain scarce. The majority of studies to date focus on hexane-extracted seed meal or mechanically pressed seed cake, as demonstrated by the following examples. A conventional alkaline extraction (pH 9.0) with acid precipitation (pH 4.5) provided a protein extract that contained 51% protein [13]. A slight modification, using an alkaline extraction pH of 11.0 and incorporating a diafiltration step prior to acid precipitation at pH 5.0, yielded a protein isolate with a significantly higher protein content of 96% [6]. Ultrasound-assisted alkaline extraction also produced a highly concentrated protein isolate with crude purity of 93.33% on a dry basis, while inducing subtle and beneficial structural modifications that enhanced functionality without causing molecular weight degradation [19]. Some studies have employed calcium chloride (pH 6.0) rather than acid for the selective precipitation of 16- and 20-kDa protein subunits, yielding concentrates with slightly reduced protein contents (76.82–77.04%) compared to traditional acid precipitation (82.04–84.32%). These low-molecular-weight fractions are generally associated with superior interfacial properties and enhanced emulsifying capacity [2,20]. Alternatively, dispersion in 0.1 mol/L sodium chloride combined with heat precipitation has been shown to yield protein isolates of 95% dry-basis purity. This process eliminates nearly all antinutrients, including glucosinolates and phytates, thereby enhancing both in vitro digestibility and techno-functional properties like foaming and emulsification [4,21]. However, studies addressing the targeted recovery of functional proteins from heat-processed industrial oilseed waste remain limited. Regarding protein chemistry, exposure to industrial thermal conditions can trigger extensive protein unfolding, while exposing internal hydrophobic domains. This promotes irreversible intermolecular cross-linking and hydrophobic aggregation, which lowers native protein solubility and extractability compared to unheated or cold-pressed counterparts [22]. Consequently, recovering proteins from thermally stressed industrial waste matrices remains difficult.

The present study examines various protein extraction treatments to valorize industrial waste from mustard (*B. juncea*) seed meal residue. The *B. juncea* residue was obtained after a sequential process of mechanical oil pressing, allyl isothiocyanate production via myrosinase catalysis, and essential oil distillation, which exposed the biomass to elevated temperatures. This experiment focused on the effects of four different extraction methods used to treat the *B. juncea* residue, including, (1) conventional alkaline extraction (CON), (2) enzyme-pretreated alkaline extraction (ENZ), (3) ultrasound-assisted alkaline extraction (US), and (4) combined enzyme and ultrasound-assisted extraction (ENZ+US) on the physicochemical, functional, and nutritional properties of the protein concentrates. Physicochemical properties were examined by determination of color, molecular mass, morphological observation, Fourier transform infrared (FTIR) spectra, and solubility. Functional properties were examined by analyzing oil absorption capacity, foaming capacity, emulsifying activity and stability, and antioxidant activities. Nutritional properties of the protein concentrates were examined by measuring the protein content and in vitro digestibility.

## 2. Materials and Methods

### 2.1. Raw Material and Chemicals

*Brassica juncea* seed meal residue, derived from sequential mechanical oil pressing and subsequent essential oil distillation was obtained from a local entrepreneur in Lamphun, Thailand. The biomass was centrifuged to collect solid by-products and dried at 50 °C in a hot air oven. The material was soaked in hexane (1:3 *w*/*v*; 3 times) to reduce fat content, pulverized, sieved through a 30-mesh screen, and stored at −20 °C prior to protein extraction. The chemical composition of the material was analyzed according to the AOAC official method [23]. All chemicals were of analytical grade and obtained from Kemaus (New South Wales, Australia), Loba Chemie (Mumbai, India), RCI Labscan (Bangkok, Thailand), Sigma-Aldrich (St. Louis, MO, USA) and Unilab Chemicals and Pharmaceuticals (Auckland, New Zealand).

### 2.2. Protein Extraction and Methods

**Figure 1:**
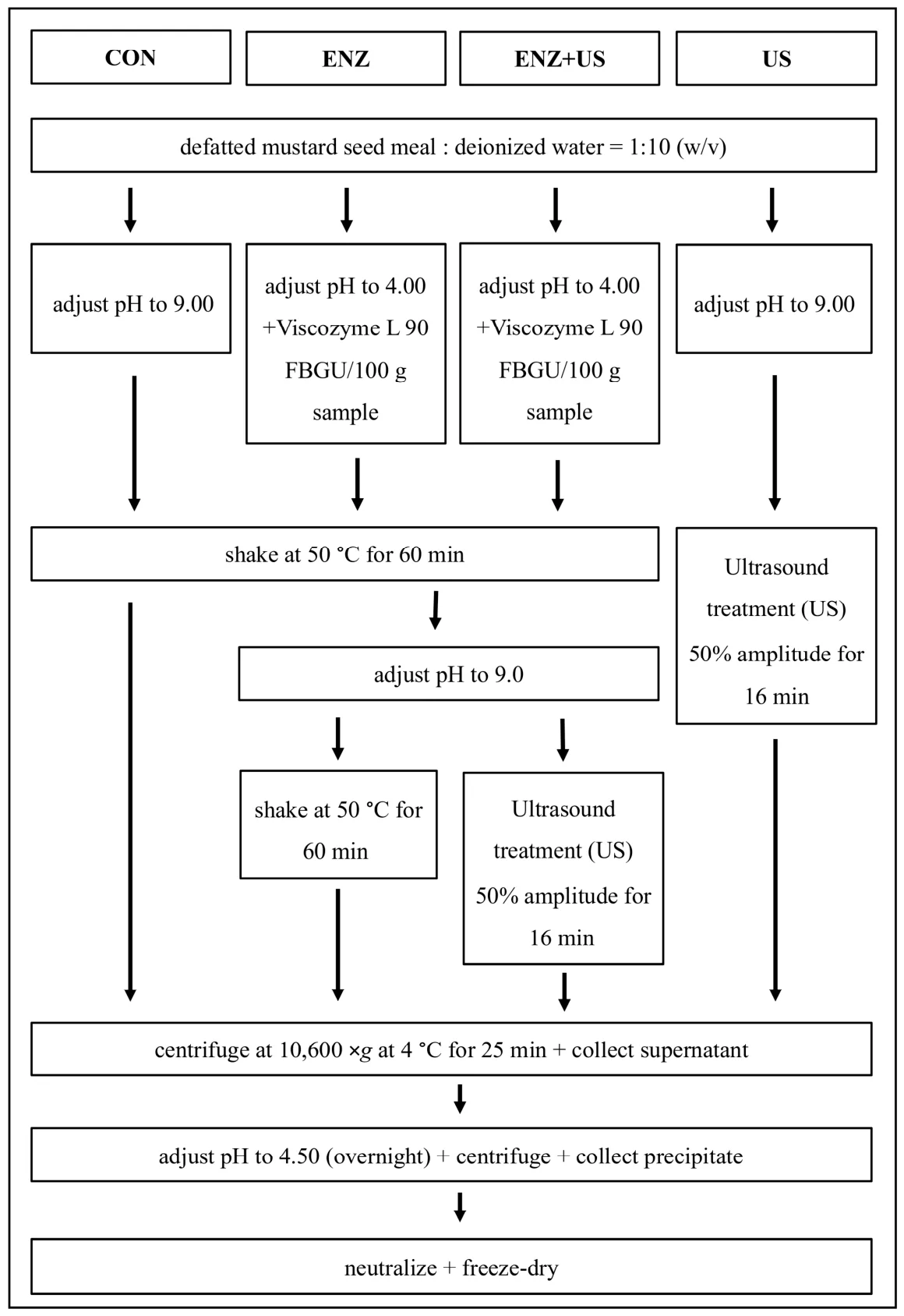
Flowchart illustrating four methods for protein extraction from mustard seed meal. CON, conventional alkaline extraction; ENZ, enzyme-pretreated alkaline extraction; ENZ+US, enzyme-pretreated and ultrasound-assisted alkaline extraction; US, ultrasound-assisted alkaline extraction.

The protein extraction experiments were divided into four distinct methods: (1) conventional alkaline extraction (CON); (2) enzyme-pretreated alkaline extraction (ENZ); (3) ultrasound-assisted alkaline extraction (US); and (4) combined enzyme and ultrasound-assisted extraction (ENZ+US). The detailed procedures for each protein extraction method (Figure 1) are described below. After an overnight protein precipitation at 4 °C, the suspension was centrifuged at 4 °C and 10,600× *g* for 25 min. The resulting precipitate was neutralized, freeze-dried, and stored at −20 °C.

#### 2.2.1. Conventional Alkaline Extraction (CON)

Conventional alkaline extraction with acid precipitation was adapted from the method of Aider et al. [4]. The material was dispersed in water at 1:10 (*w*/*v*), and the pH was adjusted to 9.00 ± 0.02 with 1 M NaOH to solubilize protein. This basic pH was selected instead of higher values to avoid protein denaturation and aggregation, which can reduce solubility at neutral and acidic pH levels [24,25]. The suspension was constantly shaken in a water bath at 50 °C for 1 h, followed by centrifugation at 4 °C and 10,600× *g* for 25 min (CR22N, Hitachi Koki Co., Ltd., Tokyo, Japan). The supernatant was filtered through filter paper (Whatman no. 4, Kent, UK) and adjusted to pH 4.50 ± 0.02 to precipitate the protein [19].

#### 2.2.2. Enzyme-Pretreated Alkaline Extraction (ENZ)

The material pretreatment using Viscozyme L prior to CON extraction was adapted from the method of Görgüç et al. [26]. The material was dispersed in water at 1:10 (*w*/*v*), and the pH was adjusted to 4.00 ± 0.02 [27] with 2 M HCl prior to the addition of Viscozyme L (90 FBGU/100 g material). The suspension was constantly shaken in a water bath at 50 °C for 1 h, followed by adjusting the pH to 9.00 ± 0.02 and further shaking at the same temperature for 1 h to solubilize the protein. The suspension was centrifuged at 4 °C and 10,600× *g* for 25 min and filtered through filter paper. The supernatant was adjusted to pH 4.50 ± 0.02 to precipitate the protein overnight at 4 °C.

#### 2.2.3. Ultrasound-Assisted Alkaline Extraction (US)

The material was dispersed in water at 1:10 (*w*/*v*) using 10 g of sample with 100 mL deionized (DI) water in a 150 mL beaker, and the pH was adjusted to 9.00 ± 0.02 with 1 M NaOH. The suspension was sonicated using a high-intensity probe-type ultrasonic processor (10 mm diameter, pulse mode: 10 s on and 10 s off, 20 kHz, 400 W, 230 V; VCX400, Sonics and Materials, Newtown, CT, USA) at 50% amplitude for 16 min, while maintaining the sample temperature below 50 °C using an ice bath. The ultrasonic probe was placed at the center of the suspension. This ultrasonic condition was evaluated in comparison with sonication parameters coupled with enzyme pretreatment (ENZ+US). The suspension was centrifuged at 4 °C and 10,600× *g* for 25 min and filtered through filter paper. The supernatant was adjusted to pH 4.50 ± 0.02 to precipitate the protein overnight at 4 °C.

#### 2.2.4. Combined Enzyme and Ultrasound-Assisted Extraction (ENZ+US)

The material was pretreated using Viscozyme L prior to US extraction. The material was dispersed in water at 1:10 (*w*/*v*), and the pH was adjusted to 4.00 ± 0.02 with 2 M HCl prior to the addition of Viscozyme L (90 FBGU/100 g material). The suspension was constantly shaken in a water bath at 50 °C for 1 h, followed by adjustment of the pH to 9.00 ± 0.02. The suspension was sonicated using a high-intensity probe-type ultrasonic processor at 50% amplitude for 16 min, while maintaining the temperature below 50 °C. These sonication parameters were selected by sequentially varying the amplitude (10–50% for 8 min) and treatment duration (4–20 min at 50% amplitude). The corresponding preliminary screening data for the alkaline-soluble extracts, evaluated based on both soluble nitrogen (N) and antioxidant activities, are provided in Supplementary Figures S1 and S2. The suspension was centrifuged at 4 °C and 10,600× *g* for 25 min and filtered through filter paper. The supernatant was adjusted to pH 4.50 ± 0.02 to precipitate the protein overnight at 4 °C.

### 2.3. Determination of Weight Yield and Protein Content of the Concentrates

The extraction weight yield (% *w*/*w*) of the mustard protein concentrates was calculated based on the dry weight of the mustard protein powder relative to the dry weight of the starting material. Protein content of the extracts was determined by the Dumas combustion method using a nitrogen analyzer (FP828, LECO, St. Joseph, MI, USA), with a nitrogen-to-protein conversion factor of 6.25 [4,6,19,20].

### 2.4. Color Measurement

The color values of freeze-dried mustard protein concentrates, expressed as lightness (L*), red-green color component (a*), and yellow-blue color component (b*), were measured using a colorimeter (CR-400, Konica Minolta, Tokyo, Japan) [28]. Total color differences (ΔE) between CON and the other protein concentrates were calculated using Equation (1).

(1) $$\Delta E=\sqrt{{\left(L^{*}\;-\;L_{0}^{*}\right)}^{2}+{\left(a^{*}\;-\;a_{0}^{*}\right)}^{2}+{\left(b^{*}\;-\;b_{0}^{*}\right)}^{2}}$$

L*, a*, and b* represent the color coordinates of the analyzed mustard protein samples, while *L*_0_*, *a*_0_*, and *b*_0_* represent those of the CON.

### 2.5. Sodium Dodecyl Sulfate Polyacrylamide Gel Electrophoresis (SDS-PAGE)

The molecular mass of mustard protein concentrates was investigated according to the method of Laemmli [29] using 10% separating gel. Protein samples were mixed with 4× loading buffer and boiled for 5 min. After samples and molecular mass protein markers (250–10 kDa; Bio-Rad Laboratories, Hercules, CA, USA) were loaded into each well and resolved, protein bands were stained overnight with Coomassie Brilliant Blue R-250.

### 2.6. Morphological Observation

The surface microstructure of mustard protein concentrates was monitored by scanning electron microscopy (SEM; JSM-IT200, Jeol, Tokyo, Japan) following the method of Karabulut et al. [30], with some modifications. The samples were gold-coated under vacuum and observed at an accelerating voltage of 15 kV under magnifications of ×200 and ×500.

### 2.7. Fourier Transform Infrared (FTIR) Spectroscopy

The transmission infrared spectra of mustard protein concentrates were measured using an FTIR spectrometer (FT/IR-4700, Jasco, Tokyo, Japan) according to the methods of Jahan et al. [19] and Kong et al. [31], with some modifications. Protein samples (10 mg) were ground into a fine powder, mixed with 1000 mg of KBr (1:100, *w*/*w*) and pressed into pellets. The spectra were monitored in a range of 4000–400 cm^−1^. Protein secondary structures were analyzed using OriginPro 2022 software (OriginLab Corporation, Northampton, MA, USA) through baseline adjustment, Fourier deconvolution, and Gaussian curve fitting across the Amide I spectral region (1700–1600 cm^−1^). The resolved sub-peaks corresponded to α-helices (1650–1658 cm^−1^), β-sheets (1610–1640 cm^−1^) β-turns (1660–1695 cm^−1^) and random coils (1640–1650 cm^−1^) [32,33]. The relative proportion of each secondary structure was determined by the portion percentage of each component.

### 2.8. Solubility at Different pHs

The protein solubility of mustard protein concentrates was examined according to the method described by Tanbamrung et al. [28] and Paksin et al. [34], with some modifications. Briefly, a protein concentration of 20 mg/mL was prepared in 0.01 M phosphate buffer solutions at various pH values (3.0, 5.0, 7.0, 9.0 and 11.0). The mixture was shaken with an orbital shaker at room temperature for 1 h, followed by centrifugation at 2490× *g* for 15 min. The protein content of the supernatant was determined by the Bradford assay using bovine serum albumin (BSA) as a standard (0.0–1.0 mg/mL) and expressed as mg/mL.

### 2.9. Oil Absorption Capacity

The oil absorption capacity of mustard protein concentrates was evaluated according to the method of Ogunwolu et al. [35]. A protein sample (100 mg) was thoroughly mixed with 1000 µL of soybean oil for 30 s using a vortex mixer (ZX3, VELP Scientifica, Usmate, MB, Italy). The mixture was then allowed to stand at room temperature for 30 min, followed by centrifugation at 13,600× *g* for 10 min. Subsequently, the sample was kept at a 45° angle for 20 min to allow the excess oil to drain. The samples were then weighed, and the oil absorption capacity was calculated using Equation (2).

(2) $$\mathrm{Oil}\;\mathrm{absorption}\;\mathrm{capacity}\;(\mathrm{mL}/g)=\frac{B}{A}$$

In the above equation, A is the weight (g) of the sample before mixing with soybean oil, and B is the volume (mL) of oil absorbed.

### 2.10. Foaming Capacity

The foaming properties of mustard protein concentrates were evaluated as described by Purdi et al. [36] and Jain and Anal [37], with some modifications. In brief, a protein sample (100 mg) was dissolved in 20 mL of DI water and homogenized (T25 digital ULTRA-TURRAX, IKA, Staufen, Germany) at 10,000 rpm for 10 min. Total volume was transferred into a graduated cylinder to measure the volume at 0 min and after 30 min. The initial and residual foaming capacities were calculated according to Equations (3) and (4), respectively. The reduction in foaming capacity after standing for 30 min was calculated by subtracting the residual value from the initial value.

(3) $$\mathrm{Initial}\;\mathrm{foaming}\;\mathrm{capacity}\;\left({FC}_{0};\;\%\right)=\frac{B-A}{A}\times 100$$

(4) $$\mathrm{Residual}\;\mathrm{foaming}\;\mathrm{capacity}\;\left({FC}_{30};\;\%\right)=\frac{C-A}{A}\times 100$$

In the above equations, A is the initial volume (mL) of a sample before homogenization, B is the total volume at 0 min following homogenization, and C is the total volume after 30 min of standing at room temperature following homogenization.

### 2.11. Emulsifying Activity and Stability

The emulsion properties of mustard protein concentrates were examined according to the methods of Aewsiri et al. [38] and Chen et al. [39], with some modifications. A protein sample (90 mg) was dissolved in 9 mL of DI water and homogenized (T25 digital ULTRA-TURRAX, IKA, Breisgau, Germany) with 1 mL of soybean oil at 13,500 rpm for 2 min. The emulsion (50 µL) was immediately collected and diluted 100-fold with 0.1% SDS (*w*/*v*) before absorbance was measured at 500 nm using a spectrophotometer (HALO XB-10, Dynamica, Hong Kong, China) at 0 min and after 10 min. The emulsion activity index and emulsion stability index were calculated using Equations (5) and (6), respectively.

(5) $$\mathrm{Emulsion}\;\mathrm{activity}\;\mathrm{index}\;\left(\mathrm{EAI};\;m^{2}/g\right)=\frac{2\;\times \;2.303\;\times \;A_{0}\;\times \;\mathrm{DF}}{L\;\times \;\phi \;\times \;C\;\times \;10^{4}}$$

(6) $$\mathrm{Emulsion}\;\mathrm{stability}\;\mathrm{index}\;(\mathrm{ESI};\;\%)=\frac{A_{10}}{A_{0}}\;\times \;100$$

A_0_ and A_10_ are the absorbances at 500 nm of diluted emulsion at 0 and 10 min, respectively. DF is the dilution factor of 100. L is the path length of a cuvette (1 cm). ϕ is the oil fraction (0.1, *v*/*v*), and C is protein concentrate concentration (0.01 g/mL).

### 2.12. Antioxidant Activities

The antioxidant properties of mustard protein concentrates were examined using Folin–Ciocalteu reducing capacity, DPPH radical scavenging, ABTS radical scavenging, ferric reducing antioxidant power (FRAP), and ferrous ion-chelating activity assays.

#### 2.12.1. Folin–Ciocalteu Reducing Capacity

The Folin–Ciocalteu reducing capacity was evaluated according to the method described by Rakariyatham et al. [40], with slight modifications. Briefly, 10 mg of a protein concentrate sample was mixed in 1 mL of DI water using a vortex mixer. A 60 μL aliquot was combined with DI water and Folin–Ciocalteu reagent at a ratio of 1:1:1 (*v*/*v*/*v*) in a microtube and agitated in the dark using an orbital shaker (Model 3015, GFL, Burgwedel, Germany) for 10 min. Next, 420 μL of 5% (*w*/*v*) Na_2_CO_3_ solution was added, and the reaction was continuously shaken in the dark for an additional 120 min. The mixture was then centrifuged at 9000× *g* for 5 min, and the absorbance of the supernatant was recorded at 765 nm (Spark, Tecan, Männedorf, Switzerland). The result was calculated from an external gallic acid calibration curve (0–200 µg/mL) and expressed as mg gallic acid equivalents (GAE)/g sample.

#### 2.12.2. DPPH Radical Scavenging Activity

The 2,2-Diphenyl-1-picrylhydrazyl (DPPH radical; DPPH•) scavenging was evaluated according to the method described by Rakariyatham et al. [40] and Tanbamrung et al. [28], with some modifications. Briefly, 10 mg of a protein concentrate sample was mixed in 1 mL of DI water. Then, a 200 µL sample was mixed with 800 µL of DPPH radical solution (200 µmol/L) in a microtube. The mixture was protected from light and shaken with an orbital shaker at room temperature for 30 min, followed by centrifugation at 9500× *g* for 5 min. The absorbance of the supernatant was measured at 517 nm. DPPH scavenging activity was calculated from an external Trolox calibration curve (0–50 µg/mL) and expressed as mg Trolox equivalents (TE)/g sample.

#### 2.12.3. ABTS Radical Scavenging Activity

The 2,2′-azino-bis(3-ethylbenzothiazoline-6-sulfonic acid) radical (ABTS•^+^) scavenging activity was evaluated according to the method described by Acharya [41] and Rakariyatham et al. [42], with some modifications. The ABTS radical cation solution was prepared by incubating 7.0 mmol/L ABTS solution with 2.45 mmol/L potassium persulfate at a 1:1 (*v*/*v*) ratio in the dark for 12–16 h. The resulting ABTS radical cation stock solution was then diluted with phosphate-buffered saline (PBS, pH 7.4) to obtain an absorbance of 0.7–0.8 at 734 nm before use. For the assay, a sample of protein concentrate (10 mg) was mixed in 1 mL of DI water using a vortex mixer. The sample (50 µL) was mixed with 950 µL of the ABTS•+ solution. The reaction was continuously shaken in the dark with an orbital shaker for 5 min. The mixture was then centrifuged at 9000× *g* for 5 min. The absorbance of the supernatant was measured at 734 nm. ABTS scavenging activity was calculated from an external Trolox calibration curve (0–200 µg/mL) and expressed as mg TE/g sample.

#### 2.12.4. Ferric Reducing Antioxidant Power

The ferric reducing antioxidant power (FRAP) assay was evaluated according to the method described by Arranz et al. [43] and Rakariyatham et al. [44], with some modifications. The FRAP working reagent was prepared by mixing 0.3 mol/L acetate buffer (pH 3.6) with 10.0 mmol/L 2,4,6-tris(2-pyridyl)-s-triazine (TPTZ), 40.0 mmol/L HCl and 20.0 mmol/L FeCl_3_ at a ratio of 10:1:1 (*v*/*v*/*v*). The mixture was then allowed to stand at 37 °C in the dark for at least 1 h before use. A sample of protein concentrate (10 mg) was mixed in 1 mL of DI water using a vortex mixer. The assay was performed by mixing 75 µL of sample with 1425 µL of FRAP reagent in a microtube. The mixture was protected from light and shaken with an orbital shaker at room temperature for 10 min, followed by centrifugation at 9500× *g* for 10 min. The absorbance of the supernatant was measured at 593 nm. The FRAP value was calculated from an external Trolox calibration curve (0–200 µg/mL) and expressed as mg TE/g sample.

#### 2.12.5. Ferrous Ion-Chelating Activity

The metal chelating activity was evaluated according to the method described by Kareem et al. [45] and Rakariyatham et al. [40], with some modification. A sample of protein concentrate (10 mg) was mixed in 1 mL of DI water using a vortex mixer. The assay was performed in a microtube by mixing 250 µL of sample with 590 µL DI water and 60 µL of 1000 µM FeSO_4_. The mixture was vortexed and allowed to stand for 2 min before adding 600 µL of 500 µM ferrozine. The mixture was then shaken in the dark with an orbital shaker at room temperature for 20 min, followed by centrifugation at 9500× *g* for 5 min. The absorbance of the supernatant was measured at 562 nm. Ferrous ion-chelating activity was calculated from an external Na_2_EDTA calibration curve (0–40 µg/mL) and expressed as mg EDTA Equivalents/g sample.

### 2.13. In Vitro Digestibility

The digestibility of mustard protein concentrates was determined by an in vitro gastrointestinal (pepsin-trypsin) digestion model according to the method of Phongthai et al. [46], with some modifications. In brief, a protein suspension (1% *w*/*v*, 100 mL) was adjusted to pH 1.5 using 3.0 mmol/L HCl. Pepsin (250 U/mg solid) was added at an enzyme-to-protein ratio of 1:100 (*w*/*w*), and the mixture was continuously shaken at 37 °C for 120 min in an incubator shaker (HH-4, Lichen Scientific, Changsha, Hunan, China). The mixture was adjusted to neutral pH with 3.0 mol/L NaOH before the addition of trypsin (10,000 BAEE U/mg protein) at an enzyme-to-protein ratio of 1:100 (*w*/*w*), and the mixture was continuously shaken at 37 °C for 120 min. The mixture was heated at 95 °C for 10 min, followed by the addition of 10% *w*/*v* trichloroacetic acid (TCA) to inactivate enzymes and precipitate undigested protein. The slurry sample was centrifuged at 6990× *g* for 10 min (CR22N, Himac, Hitachinaka, Ibaraki, Japan) to collect the precipitate, which was subsequently freeze-dried. Protein digestibility was calculated by evaluating the residual protein content in the precipitate relative to the initial protein content, which was determined via the Dumas combustion method as expressed in Equation (7).

(7) $$\mathrm{Digestibility}\;\left(\%\right)=\frac{A-B}{A}\times 100$$

In the above equation, A is the initial protein content before digestion, and B is the residual protein content after digestion.

### 2.14. Statistical Analysis

The experiments were conducted at least in triplicate, and the results were expressed as the mean ± standard deviation (SD). The data were statistically analyzed by the analysis of variance (ANOVA), followed by Duncan’s multiple range test (DMRT)/Games-Howell test using IBM SPSS statistics version 30.0 (IBM Corp., Armonk, NY, USA). Statistical significance was accepted at a level of *p* < 0.05.

## 3. Results and Discussion

### 3.1. Chemical Composition of Mustard Seed Meal Residue

The chemical composition of defatted mustard seed meal residue on a dry basis is shown in Table S1. The material had a protein content of 37.54 ± 0.17 g/100 g dry matter (DM), which is comparable to values reported in the literature (23–48 g/100 g DM), confirming its potential as an abundant source of plant-based protein [2,19,21]. The other components in the material were 35.68 ± 0.40 g/100 g DM total carbohydrate (with 7.23 ± 0.17 g/100 g DM crude fiber), 18.74 ± 0.30 g/100 g DM fat, and the minor contents of moisture (4.16 ± 0.14 g/100 g DM) and ash (3.88 ± 0.17 g/100 g DM). These values vary across studies, depending on several factors such as crop cultivar, biomass processing steps, and the specific oil extraction techniques employed [19,21].

### 3.2. Weight Yield and Protein Content of Mustard Protein Concentrates

As shown in Table 1, the weight yields of the concentrates derived from the different extraction methods (CON, ENZ, US, and ENZ+US) were not significantly different, ranging from 0.80–1.14% *w*/*w*. This corresponds to a protein recovery of 1.65–2.50% *w*/*w*, calculated based on the initial protein content of the raw material. This low extraction yield may be explained by partial protein denaturation during industrial processing, which compromises protein solubility during the extraction process. Specifically, the raw material underwent mechanical oil pressing, followed by myrosinase-catalyzed isothiocyanate production (at optimal temperatures of 30–60 °C, depending on the biological source [47,48,49]) and high-temperature essential oil distillation [50]. Previous studies on rapeseed and pumpkin seed meals have also demonstrated lower protein extraction yields when the raw materials were exposed to high-temperature processes, such as conventional screw pressing, roasting, boiling, and desolventizing–toasting [51,52,53,54]. High temperatures disrupt the native structure of major storage proteins, causing a physical dissociation of high-molecular-weight proteins and a high degree of disulfide bond cleavage of cruciferin heterodimers into smaller chains. This structural unfolding unmasks non-polar, hydrophobic residues previously buried in the interior of the protein molecules, altering the natural hydrophobicity/hydrophilicity balance and rendering the proteins significantly less soluble in aqueous media [51,55]. In addition to physical denaturation, low-moisture heating conditions (particularly during mechanical pressing) can promote a Maillard reaction, generating reactive advanced products like glyoxal and methylglyoxal. These compounds react with basic amino acid residues (such as lysine, arginine, and tryptophan) to induce protein-to-protein cross-linking that lowers protein solubility and thus inhibits extractability [51,56].

The protein contents (Table 1) of the resulting concentrates differed significantly (*p* < 0.05), descending in the order of CON (82.31 ± 0.24%) > US (77.88 ± 0.31%) > ENZ (74.96 ± 0.34%) > ENZ+US (70.67 ± 0.20%). The lower protein content observed in the individual or combined treatments of Viscozyme L and ultrasound can be attributed to the underlying mechanisms of cell-wall-degrading enzymes and acoustic cavitation. These processes can liberate not only intracellular proteins but also non-proteinaceous cell wall components that are co-extracted alongside the target proteins, thereby diluting the final protein content [14,57,58]. Similar trends have been reported for both duckweed and brewer’s spent grain protein extractions [59,60].

### 3.3. Physicochemical Properties of Mustard Protein Concentrates

#### 3.3.1. Color

**Figure 2:**
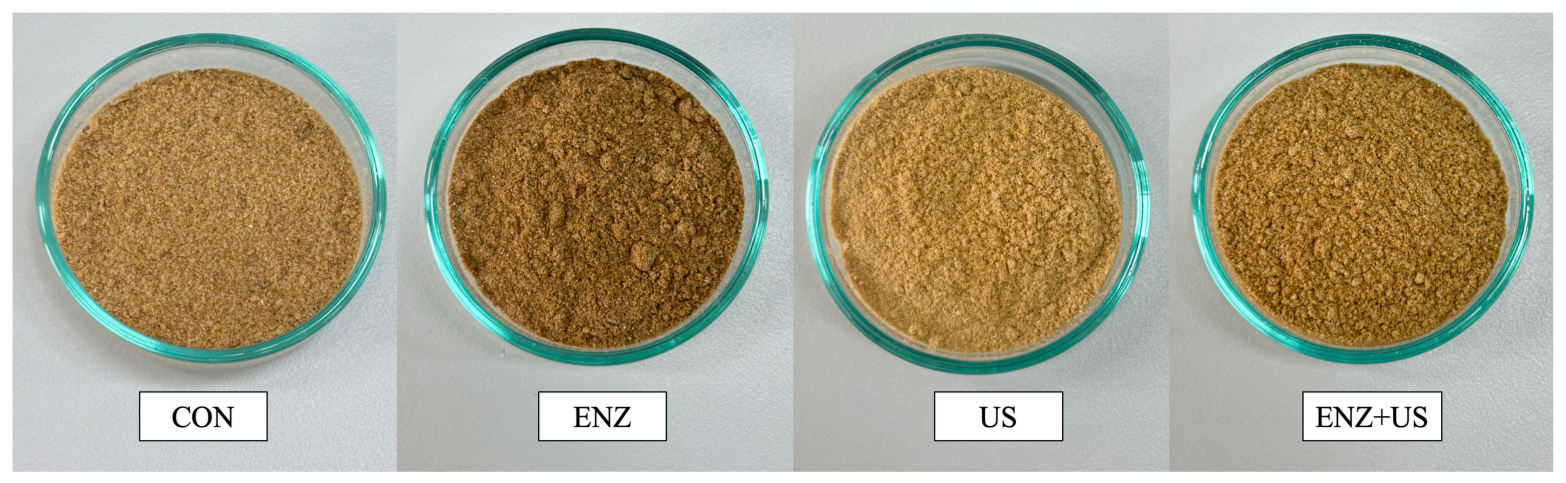
Mustard proteins extracted by different methods. CON, conventional alkaline extraction; ENZ, enzyme-pretreated alkaline extraction; US, ultrasound-assisted alkaline extraction; ENZ+US, enzyme-pretreated and ultrasound-assisted alkaline extraction.

The physical appearance of the mustard protein concentrates is shown in Figure 2, which correlates with the instrumental color parameters obtained via the CIELAB system (Table 1). The color profile revealed that the protein concentrates possessed L*, a*, and b* values of 54.29–64.36, 5.73–6.57 and 20.13–23.88, respectively. This indicates a dominant yellow spectrum with low redness chromaticity, likely originating from co-precipitation of inherent carotenoids and phenolic compounds native to the mustard seed alongside the proteins [61,62,63].

Compared to the CON group, the independent applications of Viscozyme L (ENZ) darkened the protein concentrate, as evidenced by a significant decrease in L* from 59.46 ± 0.48 to 54.29 ± 3.06, and a decrease in b* from 23.88 ± 0.50 to 20.13 ± 1.28, while the a* value remained statistically unchanged, leading to the ΔE of 7.14 ± 0.42. Since the ΔE value is greater than 3.0 units, the color difference is perceptible to the human eye [64]. The decline of b* is likely due to the release of plant cell wall components by Viscozyme L, which acts as a physical bulking agent that dilutes the chroma of the native yellow pigments. However, during enzymatic incubation, Viscozyme L can simultaneously disrupt cell walls and release residual endogenous polyphenol oxidases (PPOs). These enzymes oxidize phenolics into reactive quinones, which subsequently cross-link with proteins to form dark complexes, thereby decreasing the lightness (L*) of the concentrate [63,65,66,67,68,69,70].

The ENZ+US treatment, showing the lowest ΔE (3.26 ± 0.20), did not significantly change the values of L* (57.38 ± 1.44), a* (6.15 ± 0.27), and b* (22.62 ± 1.00) compared to CON. This can be attributed to acoustic cavitation-induced degradation of inherent bioactives and pigments, as well as the inactivation of PPO, leading to lower color intensity in the ENZ+US protein concentrate compared to that of ENZ [71,72]. Studies have revealed the potential of ultrasonication in inactivating PPO, as acoustic cavitation generates reactive hydroxyl radicals (•OH) and hydrogen peroxide (H_2_O_2_) in the medium. These species oxidize amino acid residues and disrupt disulfide linkages, leading initially to protein dissociation, followed by aggregation and secondary and/or tertiary structural reorganization at higher acoustic intensities [73]. Furthermore, reactive species generated during acoustic cavitation can induce the oxidative degradation of phenolics and carotenoids, the extent of which is governed by ultrasound amplitude and exposure duration [74]. Corresponding data were obtained from the single ultrasound treatment (US), where L* (64.36 ± 0.36) was the highest. Consistent with these findings, the integration of ultrasonication during the extraction of proteins from lambsquarters seeds and oats similarly enhanced the lightness profile of the resulting concentrates [75,76]. In addition, Villacís-Chiriboga et al. [76] observed that the lightness of the protein extract was sensitive to processing order, varying significantly depending on whether Viscozyme L hydrolysis preceded or followed ultrasonication.

#### 3.3.2. Molecular Mass

SDS-PAGE analysis was performed to investigate the effect of extraction methods on molecular masses of polypeptides in protein concentrates. Supplementary Figure S3 shows polypeptide bands separated by mass under a non-reducing condition. The NaCl-alkaline extracted sample (where 1 M NaCl was added to the alkaline solution during extraction) is included solely in this experiment to serve as a comparative reference. Major bands were observed at approximately 15 kDa, within 15–20 kDa, 25–37 kDa, and 37–50 kDa. In addition, a minor band with a higher molecular mass of 75 kDa was observed only in non-saline-containing systems (CON, US, ENZ, ENZ+US). The results correspond to the findings of Aluko et al. [2] and Rao and Rao [77], who reported molecular masses of protein from *B. juncea* in the ranges of 12–80 kDa and 11–70 kDa, respectively. Previous studies in Brassicaceae oilseeds including *B. juncea* reported that the seed proteins were predominantly composed of hexameric 11S or 12S cruciferin (globulin; 300–360 kDa) and monomeric 1.7S or 2S napin (albumin; 15–18 kDa). Each subunit of the hexameric cruciferin consists of an acidic α-chain (~30 kDa) and a basic α-chain (~20 kDa) held together by a disulfide bond. Under non-reducing SDS-PAGE conditions, these linked chains migrate together as a single complex at 48–56 kDa but resolve into separate bands between 18 and 53 kDa upon reduction. In contrast, the monomeric napin (~14–17 kDa) consists of a small light chain (4–6 kDa) and a large heavy chain (10–12 kDa) stabilized by four disulfide bonds [1,11,78,79,80,81].

Notably, the CON and US treatments exhibited more pronounced band intensities within the 37–50 kDa range. In contrast, the ENZ and ENZ+US treatments showed greater intensity in the 15–20 kDa region, whereas the comparative saline-alkaline extraction displayed its highest intensities in both the 15–20 kDa region and at the 15 kDa band. The results indicate that the extraction methods, especially the type of solvent, influenced polypeptide composition of the protein concentrates. Alkaline extraction can solubilize both globulin and albumin. However, subsequent acid precipitation at pH 4.0–5.0 aligns more closely with the pI of plant globulins (estimated at 7.2 for cruciferin), leading to selective precipitation that yields protein concentrates where globulin predominates over albumin or napin [14,82], as demonstrated in the CON and US treatments. In contrast, napin maintains a net positive charge under acidic conditions due to its high pI (≈10–11). This charged state may facilitate electrostatic complexation with cell-wall poly-, oligo-, or short-chain saccharides liberated during Viscozyme L pretreatment, resulting in co-precipitation as evidenced by the increased band intensities observed in the ENZ and ENZ+US treatments [82,83]. In saline-containing systems, napin exhibited higher band intensities than cruciferin. Incorporating sodium chloride into the alkaline medium can alter the ionic environment and prompt protein conformational changes. Specifically, the salt drives a ‘salting-in’ effect on cruciferin, stabilizing its structure and increasing solubility at pH 5.0–7.0 [79,81]. Conversely, sodium chloride modulates the pI of napin by inducing a downward shift toward the acidic range (3.5–3.9). Driven by the preferential adsorption of chloride ions (Cl^−^) onto napin’s protonated surface, this mechanism may promote aggregation and accounts for the prominent napin bands resolved on the non-reducing SDS-PAGE [84].

#### 3.3.3. Morphology

**Figure 3:**
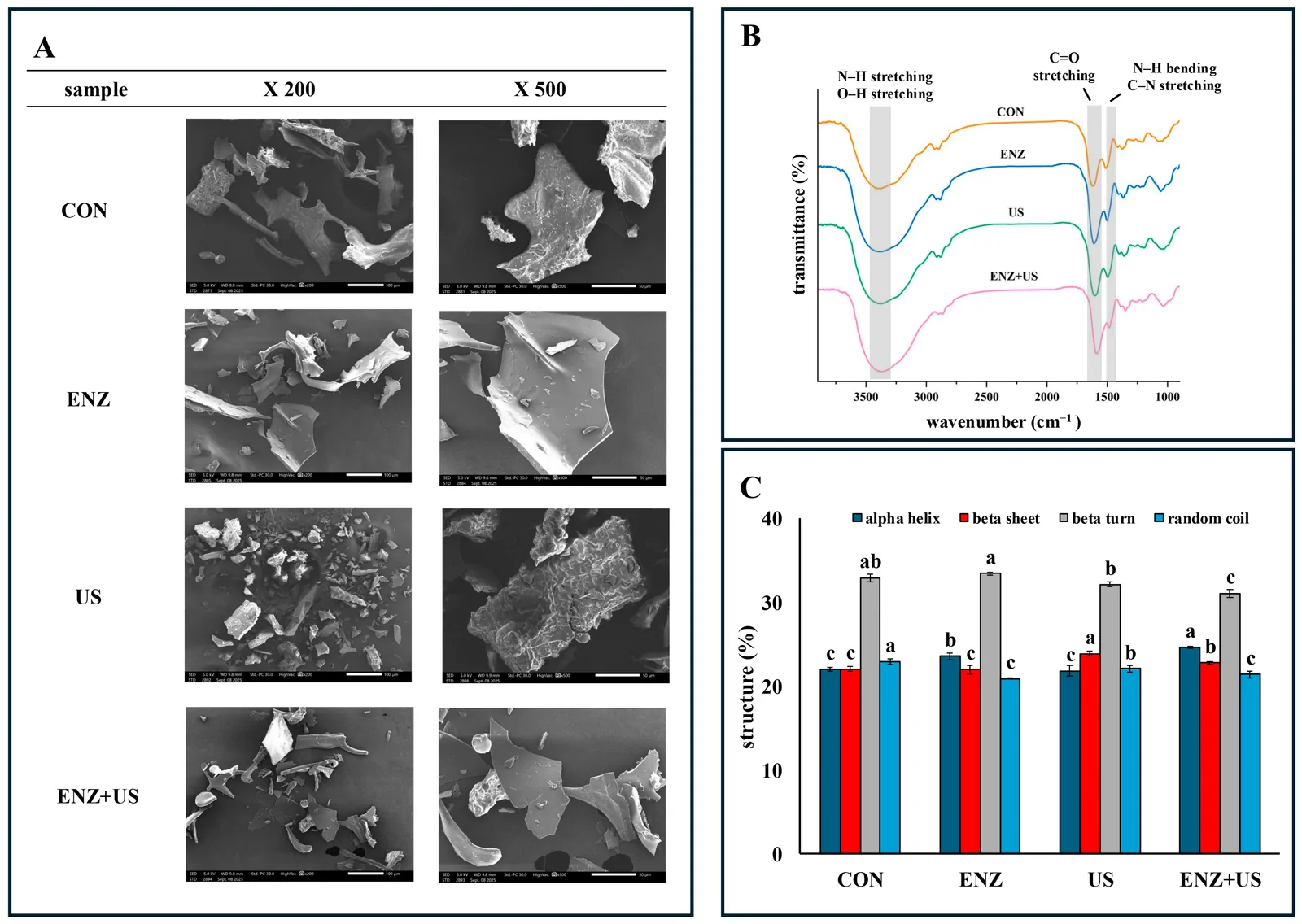
Scanning electron micrographs (**A**), FTIR spectra (**B**) and secondary structures (**C**) of mustard proteins extracted by different methods. CON, conventional alkaline extraction; ENZ, enzyme-pretreated alkaline extraction; US, ultrasound-assisted alkaline extraction; ENZ+US, enzyme-pretreated and ultrasound-assisted alkaline extraction. Values of secondary structure are expressed as mean ± SD. ^a–c^ letters demonstrate significant differences in each structure among samples (*p* < 0.05).

The SEM images of mustard protein concentrates obtained from different extraction methods are displayed in Figure 3A. The proteins were irregularly shaped, varied in size, and exhibited distinctly different surface morphologies. Specifically, proteins extracted using the CON method exhibited a plate-like appearance with a noticeably rough surface. This observation aligns with prior studies on other plant proteins such as hemp protein isolate and pea pod protein concentrate extracted via similar alkaline extraction with acid precipitation methods [28,30]. Compared to CON, the ENZ treatment yielded a smoother surface, which is likely due to the activity of Viscozyme L breaking down cell-wall polysaccharides into short-chain fragments. These carbohydrate fragments can co-precipitate with the proteins, creating a unified biopolymer matrix that minimizes surface roughness upon drying [85]. A similar morphological pattern has been observed in proteins extracted from moringa seeds, common bean seeds, and pea pods that were subjected to Viscozyme L pretreatment prior to extraction [30,86,87]. In contrast, the US extraction method produced a more fragmented protein matrix with a highly rugged surface. This rough topography is likely driven by intense acoustic cavitation, which fractures the protein networks and reduces particle dimensions through localized mechanical breakdown [86,88,89,90]. While individual ENZ and US treatments resulted in uniform, distinct microstructures, the combined ENZ+US treatment exhibited a mixed morphology containing both smooth and rough surfaces. This suggests that while ultrasound mechanically fragments the bulk protein aggregates, associative protein-saccharide interactions may remain active, continuing to assemble and smooth out localized interfaces upon drying.

#### 3.3.4. FTIR Spectra

Structural characteristics of protein concentrates were analyzed from FTIR spectra (Figure 3B). The major bands relating to the protein backbone include amide A (3450–3300 cm^−1^; reflects N–H stretching and hydrogen bonding), Amide I (1700–1600 cm^−1^; reflects C=O stretching), and Amide II (1600–1500 cm^−1^; reflects N–H bending and C–N stretching) [91,92]. In the CON sample, the peaks of amide A, Amide I and Amide II were at 3404.33, 1626.29, and 1523.11, respectively. Amide A peak intensities increased noticeably across the ENZ, US, and ENZ+US treatments. This spectral alteration suggests intensified hydrogen bonding interactions following either individual or combined Viscozyme L and ultrasonication processing, reflecting enhanced intra- and/or intermolecular hydrogen bonding networks within the protein concentrates [93]. Since the 3200–3700 cm^−1^ region, which overlaps with the Amide A band, is also associated with hydrogen bonded O–H stretching, the increased intensity of this broad peak may reflect the abundance of hydroxyl groups [28]. These groups were likely released from the plant matrix and co-extracted alongside the protein concentrates. Additionally, hydrogen bonding to the C=O group and N–H group could also affect their vibrations, leading to shifting of amide peaks to lower wavenumber values [39,91]. Relative to the CON, a pronounced shift toward lower frequencies was observed for the Amide I and Amide II bands. The magnitude of this shift decreased in the order of ENZ (at 1616.59, and 1511.48) followed by US (at 1606.70, and 1508.35) and ENZ+US (at 1603.03, and 1500.82). These data suggest that different extraction methods, including Viscozyme L pretreatment and/or ultrasonication, affect protein conformation, resulting in increased hydrogen bond formation [94].

An analysis of the Amide I band, which is the most sensitive region for protein secondary structure [95], demonstrates that β-turn was the main structure (31.08–33.67%) in mustard protein (Figure 3C). In comparison to CON, ENZ had a significantly increased α-helix structure from 22.03 ± 0.26% to 23.60 ± 0.40%, while US had a significantly increased β-sheet structure from 22.09 ± 0.32% to 23.91 ± 0.25%. The ENZ+US treatment exhibited the effects of both Viscozyme L pretreatment and ultrasonication by increasing the α-helix (24.69 ± 0.12%) and β-sheet (22.80 ± 0.20%) structures while decreasing the β-turn structure (31.08 ± 0.41%). Notably, all treatments significantly reduced the disordered random coil conformations while simultaneously increasing the proportions of more ordered secondary structures. These results correspond to previous studies reporting that ultrasonic treatment of protein isolates from soybean, buckwheat, and chickpea increased β-sheet structure while decreasing random coil conformations [88,96,97]. Another study [98] reported a similar decrease in random coils, though it resulted in an increase in α-helix structures rather than β-sheets. This is likely due to acoustic cavitation affecting interactions and bonds in native protein structures, leading to conformational changes during renaturation.

#### 3.3.5. Solubility at Different pHs

**Figure 4:**
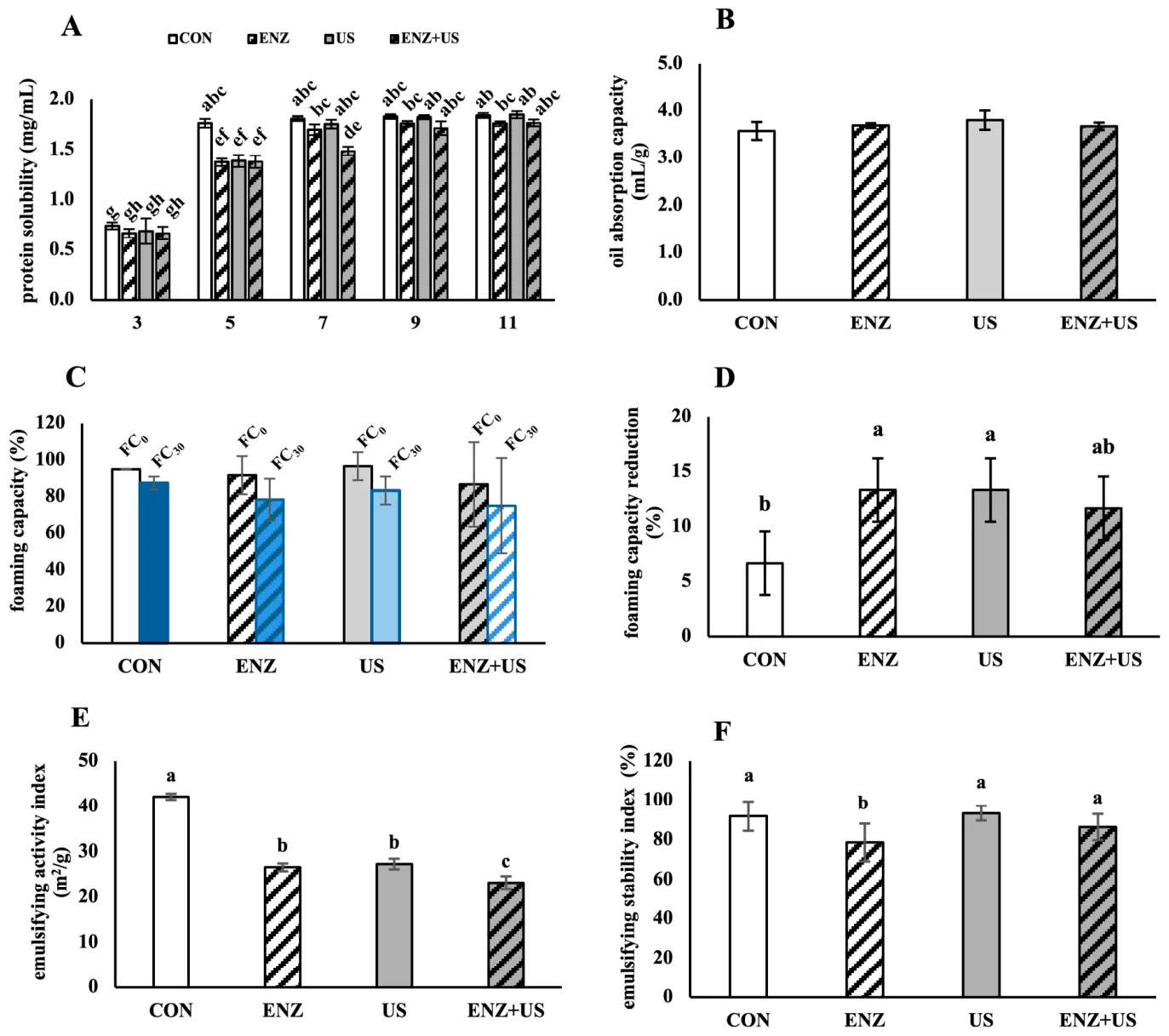
Solubility (**A**), oil absorption capacity (**B**), foaming capacity (**C**), foaming capacity reduction (**D**), emulsifying activity index (**E**), and emulsifying stability index (**F**) of mustard proteins extracted by different methods. CON, conventional alkaline extraction; ENZ, enzyme-pretreated alkaline extraction; US, ultrasound-assisted alkaline extraction; ENZ+US, enzyme-pretreated and ultrasound-assisted alkaline extraction. Values are expressed as mean ± SD. ^a–h^ letters demonstrate significant differences among samples (*p* < 0.05).

Since protein solubility is a fundamental property influencing other functions, including emulsifying and foaming capacities, antioxidant capacities, and bioavailability, this study analyzed the solubility of mustard protein concentrates as a function of pH in the range of 3.0–11.0. As shown in Figure 4A, across the analyzed pH range, the maximum solubility of all protein concentrates occurred at pH 11.0 (ranging from 1.76–1.85 mg/mL), whereas the minimum solubility was observed at pH 3.0, ranging from 0.67–0.74 mg/mL. As oilseeds contain a relatively high amount of phytic acid, which can form insoluble protein–phytate complexes during pH 4.5 precipitation [81,99,100,101], this may explain why the protein solubility range of 0.67–1.85 mg/mL was observed in this study. However, the observed trends of reduced solubility near the pIs and higher solubility under alkaline conditions still align with previous studies on plant proteins including mustard proteins [4,19,99,102,103].

The solubility profiles of the protein concentrates (CON, ENZ, US, and ENZ+US) correspond with their protein composition observed via SDS-PAGE (Supplementary Figure S3), where these extracts were predominantly high in globulins. This profile directly dictates solubility, as globulin and albumin storage fractions possess different hydration characteristics and disparate pI values [14,82,104]. Across the pH range of 3.0 to 11.0, CON maintained a higher solubility profile than the single or combined assisted extractions (ENZ, US, and ENZ+US). While these protein concentrates exhibited improved solubility as a function of increasing pH, structural modifications induced by Viscozyme L, ultrasonication, or both may have promoted partial hydrophobic exposure or altered structural conformations during processing [97,105], leading to reduced solubility relative to CON.

### 3.4. Functional Properties of Mustard Protein Concentrates

#### 3.4.1. Oil Absorption Capacity

Oil absorption capacity reflects the structural affinity of proteins to bind lipids, thereby serving as a critical determinant of food textural attributes such as mouthfeel and flavor perception as well as macroscopic emulsification functionalities [106]. As shown in Figure 4B, CON, ENZ, US, and ENZ+US exhibited oil absorption capacity in the range of 3.57–3.80 mL/g. The oil-binding capacity of proteins is determined by a complex interplay of structural attributes, including molecular size, flexibility, and degree of denaturation, alongside the distribution of surface-exposed nonpolar amino acid chains [107,108,109]. In addition, these results can be traced to their higher cruciferin-to-napin ratios in the concentrates extracted by non-saline methods (Supplementary Figure S3). In conjunction with the study by Perera et al. [110], who demonstrated that cruciferin exhibits higher surface hydrophobicity than napin across all pH ranges, these findings explain how this elevated non-polarity imparts a significantly higher lipid-binding affinity to the concentrates extracted by non-saline methods [19,111].

#### 3.4.2. Foaming Capacity

In general, protein foaming properties depend on the molecule’s capacity to rapidly adsorb to and stabilize the air–water interface. This behavior is governed by a combination of intrinsic characteristics, including protein composition, surface hydrophobicity, and conformational flexibility, as well as system variables such as concentration, pH, temperature, ionic strength, and intermolecular interactions [14,112,113]. The foaming capacity (foamability) of mustard protein concentrates, representing the total volume increase by the proteins at 0 min (initial foaming capacity; FC_0_) and 30 min (residual foaming capacity; FC_30_) after homogenization, is displayed in Figure 4C. CON, ENZ, US, and ENZ+US exhibited substantial FC_0_ values, ranging from 86.67–96.67%. The foaming capacity or the foam expansion of all samples decreased after 30 min to FC_30_ values ranging from 75.00–87.50%, where CON demonstrated the lowest capacity reduction of 6.67%, followed by ENZ+US (11.67%), and ENZ ≈ US (13.33%), respectively (Figure 4D). In addition, a decrease in foam density was observed alongside the reduction in foaming capacity, which could be explained by the protein content in the concentrates (Table 1) where CON possessed the highest content. Because the interfacial and foam properties of protein depend on bulk viscosity and protein concentration [93,113], the lower protein content in certain samples suggests the presence of co-extracted non-protein impurities. These components can compromise the viscosity of the aqueous phase, leading to a weaker, less stiff film at the air–water interface that fails to protect the bubbles from draining [14].

#### 3.4.3. Emulsifying Activity and Stability

As emulsions comprise two immiscible liquids with one phase dispersed as droplets within the other, such as an oil-in-water system, the efficacy of a protein stabilizer depends on its adsorption rate at the droplet surface, which varies primarily by the intrinsic physicochemical properties of the protein, including molecular size, protein composition, conformation, surface hydrophobicity, and solubility [14,114]. The emulsifying activity index (EAI) of mustard protein concentrates, representing the ability of protein to form an emulsion, is displayed in Figure 4E. CON showed an EAI of 42.15 ± 0.69 m^2^/g, which was similar to values reported in a previous study for protein concentrates from the same plant (*B. juncea*), ranging from 39.85–44.47 m^2^/g [20]. The magnitude of these EAI values reflects the predominant protein, cruciferin (Supplementary Figure S3). Its larger molecular size retards interfacial diffusion, and its balanced hydrophobic/hydrophilic domain distribution diminishes its ability to anchor molecules of differing polarities relative to napin [115]. Compared with the other protein concentrates, CON extracted proteins possessed significantly higher EAI than ENZ, US, and ENZ+US (23.26–27.36 m^2^/g) extracted proteins. These ENZ, US, and ENZ+US samples, which likely contained co-precipitated non-protein impurities, may have formed insoluble protein complexes. These interactions may reduce water solubility at neutral pH relative to the CON treatment (Figure 4A), inducing structural modifications and suppressing zeta-potential, which ultimately diminished overall emulsifying activity [116]. These findings are supported by previous studies including plant proteins extracted via carbohydrase- or ultrasound-assisted methods [17,105,117,118,119,120].

The emulsifying stability index (ESI) of mustard protein concentrates, representing the stability of the emulsion over a specific period, is displayed in Figure 4F. The concentrates extracted by the CON and US treatments possessed higher ESI values (91.98–93.44%) than ENZ+US (86.41 ± 6.62%) and ENZ (78.69 ± 9.50%), respectively. These findings are supported by SEM analysis (Figure 3A), which revealed smoother particle surfaces across all Viscozyme L-assisted treatments (ENZ and ENZ+US) compared to the other treatments without the enzyme. This smooth surface morphology likely reflects a shell of co-extracted, soluble cell wall components enveloping the protein. This layer may function as a steric barrier that impairs protein conformational unfolding and interfacial network formation, thereby diminishing overall emulsion stability over time [121].

#### 3.4.4. Antioxidant Capacity

**Figure 5:**
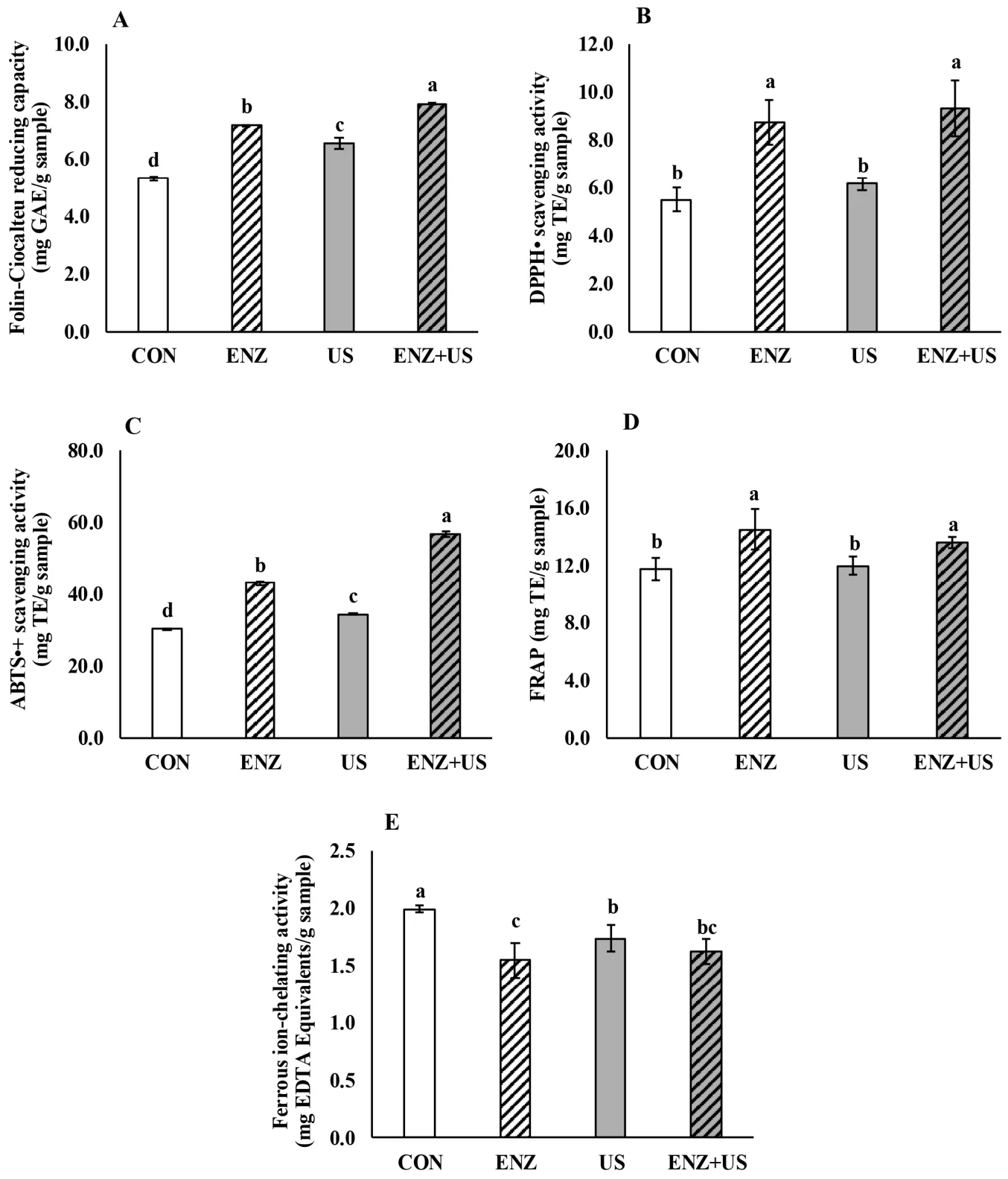
Folin–Ciocalteu reducing capacity (**A**), DPPH radical scavenging activity (**B**), ABTS radical scavenging activity (**C**), ferric reducing antioxidant power (FRAP) (**D**), and ferrous ion-chelating activity (**E**) of mustard proteins extracted by different methods. CON, conventional alkaline extraction; ENZ, enzyme-pretreated alkaline extraction; US, ultrasound-assisted alkaline extraction; ENZ+US, enzyme-pretreated and ultrasound-assisted alkaline extraction. Values are expressed as mean ± SD. ^a–d^ letters demonstrate significant differences among samples (*p* < 0.05).

The antioxidant activities of mustard protein concentrates were evaluated using the following five methods. While the Folin–Ciocalteu reducing capacity, DPPH and ABTS radical scavenging, and ferric reducing antioxidant power (FRAP) assays assess hydrogen atom transfer (HAT) and/or single electron transfer (SET) mechanisms, ferrous ion-chelating activity serves as a complementary measure of secondary, or a preventive antioxidant mechanisms [122,123]. Across the first four assays (Figure 5A–D), a consistent trend was observed. The concentrates extracted by Viscozyme L-assisted methods (ENZ and ENZ+US) displayed significantly higher antioxidant capacity compared to their non-enzyme-assisted counterparts. Specifically, values ranged from 7.20–7.95 mg GAE/g for Folin–Ciocalteu reducing capacity, 8.75–9.33 mg TE/g for DPPH radical scavenging, 42.97–56.86 mg TE/g for ABTS radical scavenging, and 13.59–14.49 mg TE/g for FRAP. This antioxidant trend is inversely correlated with the protein content of the concentrates (Table 1), suggesting that co-extracted non-protein compounds digested and released by Viscozyme L contributed significantly to the overall antioxidant capacity [124]. However, single US treatment exhibited lower antioxidant activities than ENZ and ENZ+US, though the values remained higher than those of treatments the CON treatment. This may be attributed to the generation of reactive species during ultrasound-assisted extraction, wherein acoustic cavitation induces the sonolysis of water molecules during bubble collapse, producing hydroxyl radicals and hydrogen peroxide, which can oxidize antioxidant molecules in the extract [125,126,127].

This finding provides evidence for the dual role of ultrasonication. The enhanced extraction of bioactive compounds occurs simultaneously with sonochemically induced free radical formation driven by acoustic cavitation. These free radicals act as a double-edged sword, significantly increasing visual lightness (L*; Table 1) through pigment degradation and enzyme inactivation [73,74], while concurrently decreasing antioxidant capacity. Therefore, while single US treatment is highly effective for improving the lightness of the concentrate, this benefit comes at the cost of oxidative degradation. To overcome this trade-off, hybrid approaches such as the ENZ+US treatment are necessary, as they facilitate a robust release of antioxidant compounds, including phenolics, that quantitatively compensate for sonochemical oxidative losses, ultimately yielding a product exhibiting both high lightness and superior antioxidant activity.

The final assay, ferrous ion-chelating activity, revealed an opposite trend compared to the other antioxidant parameters. The CON treatment, containing the highest protein content (82.31% *w*/*w*; Table 1), exhibited the highest iron chelating activity (1.99 ± 0.03 mg EDTA equivalents/g) compared to US (1.73 ± 0.12 mg EDTA equivalents/g), ENZ+US (1.62 ± 0.11 mg EDTA equivalents/g), and ENZ (1.54 ± 0.15 mg EDTA equivalents/g), respectively. These data suggest that the co-precipitated bioactives responsible for enhancing the antioxidant capacity of the protein concentrates via hydrogen atom transfer and single electron transfer mechanisms in treated extraction methods (ENZ, US and ENZ+US) did not exhibit significant iron-chelating activity. Previous studies have shown that alkaline extraction of Brassicaceae oilseed proteins can co-extract bioactive compounds possessing metal chelating activity, such as tannins, sinapic acid and/or sinapine [6,128,129,130,131]. This co-extraction may account for the higher ferrous ion-chelating capacity observed in CON compared to its processed counterparts, which contained a higher proportion of other types of co-precipitates.

### 3.5. Nutritional Properties of Mustard Protein Concentrates

**Figure 6:**
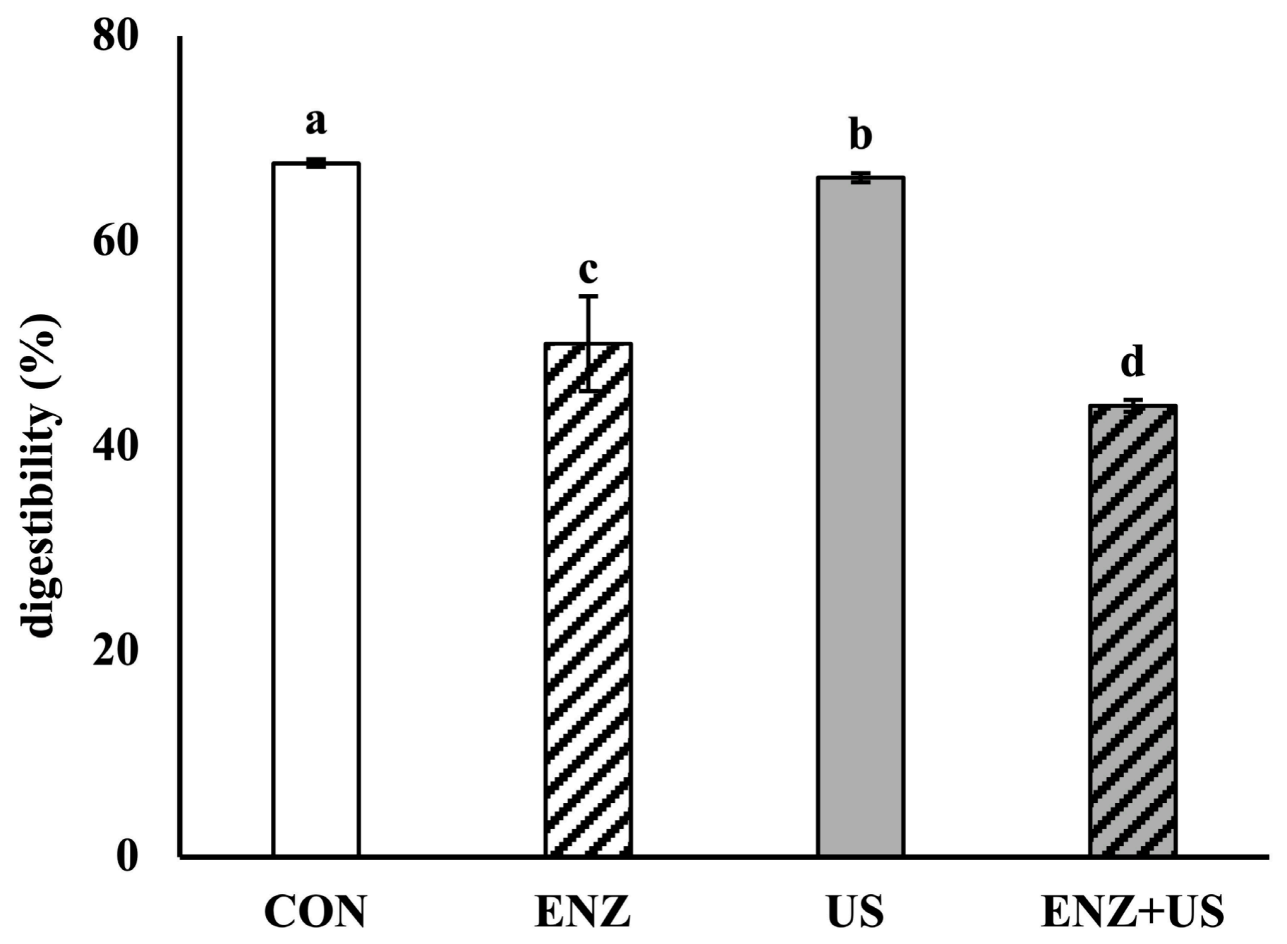
In vitro digestion of mustard proteins extracted by different methods. CON, conventional alkaline extraction; ENZ, enzyme-pretreated alkaline extraction; US, ultrasound-assisted alkaline extraction; ENZ+US, enzyme-pretreated and ultrasound-assisted alkaline extraction. Values are expressed as mean ± SD. ^a–d^ letters demonstrate significant differences among samples (*p* < 0.05).

The results for the in vitro digestibility of mustard protein concentrates are shown in Figure 6. The CON treated extract possessed the highest digestibility at 67.63 ± 0.40%, which was similar to a previous study reporting pepsin digestibility of protein from yellow mustard cake before the removal of antinutritional compounds at 77.43% [132]. Protein concentrate extracted by the US method had the second highest digestibility at 66.19 ± 0.43%. This may be attributed to the high intensity ultrasonication, which partially induces protein aggregation and forms dense, compact structures that exhibit reduced susceptibility to enzymatic hydrolysis compared to CON [88]. In addition, a reduction in key anti-nutritional factors, including phytate, phenolics (e.g., sinapine), and glucosinolate breakdown products (i.e., allyl isothiocyanate), has been shown to contribute to enhanced pepsin digestibility [132,133,134]. Because the utilization of Viscozyme L in extraction methods can lead to hydrolysis of fibrous portions of cell walls and the release of antinutrients [15,135,136], protein concentrates derived from ENZ and ENZ+US may co-precipitate these antinutrients, thus demonstrating significantly lower digestibility, in the range of 43.95–50.02%, than their non-enzyme-treated counterparts. Overall, cumulative data on physical, functional, and digestive properties suggest that the extraction methods yield distinct combinations of non-protein co-precipitates that serve dual roles. While these co-precipitates, especially from the ENZ and ENZ+US methods, enhance antioxidant activity through hydrogen or electron donation, they concurrently decrease the visual lightness, aqueous solubility, interfacial functionality, and digestibility of the final matrix, underscoring the need to balance bioactive recovery with techno-functional preservation.

## 4. Conclusions

This study demonstrates that the compositional, physicochemical, functional, and nutritional properties of mustard seed meal protein concentrates can be tailored by selecting appropriate extraction methods. Each evaluated method, i.e., conventional alkaline extraction (CON), enzyme-pretreated alkaline extraction (ENZ), ultrasound-assisted alkaline extraction (US), and enzyme-pretreated and ultrasound-assisted alkaline extraction (ENZ+US), offers distinct advantages based on the targeted product properties. Specifically, enzymatic treatments (ENZ and ENZ+US) utilizing Viscozyme L effectively hydrolyzed cell-wall polysaccharides, resulting in smoother protein morphologies and the co-extraction of non-protein bioactives. This structural breakdown significantly enhanced the antioxidant capacities of the concentrates. In contrast, although single ultrasound extraction (US) may generate reactive species that reduce antioxidant capacity, it increased the visual lightness (L*) and maintained in vitro digestibility at a level comparable to the control. The results show that among the tested laboratory scale treatments, CON extraction remains the optimal choice for applications prioritizing functional performance, as it exhibited the highest protein solubility across the evaluated pH range and yielded the highest emulsifying activity index. In summary, this research highlights the potential of transforming mustard oilseed waste into high-value functional ingredients by aligning extraction protocols with desired characteristics. However, low extraction yields present a significant bottleneck to commercialization. Achieving industrial and economic feasibility will rely heavily on future yield optimization to drive the sustainable use and recovery of these plant-based proteins.

## Figures and Tables

**Table 1:** Weight yield, protein content and color of mustard protein concentrates.

Sample	Protein Concentrate Weight Yield ^ns^ (% *w*/*w*)	Protein Content (% *w*/*w*)	L*	a*	b*	ΔE
CON	1.14 ± 0.20	82.31 ^a^ ± 0.24	59.46 ^ab^ ± 0.48	6.57 ^a^ ± 0.12	23.88 ^a^ ± 0.50	-
ENZ	0.86 ± 0.28	74.96 ^c^ ± 0.34	54.29 ^c^ ± 3.06	6.24 ^a^ ± 1.17	20.13 ^b^ ± 1.28	7.14 ^a^ ± 0.42
US	0.80 ± 0.15	77.88 ^b^ ± 0.31	64.36 ^a^ ± 0.36	5.73 ^b^ ± 0.07	23.86 ^a^ ± 0.10	5.39 ^b^ ± 0.57
ENZ+US	0.97 ± 0.10	70.67 ^d^ ± 0.20	57.38 ^bc^ ± 1.66	6.15 ^a^ ± 0.27	22.62 ^a^ ± 1.00	3.26 ^c^ ± 0.20

Data is presented as mean ± SD (n ≥ 3). Values with different letters differ significantly according to Duncan’s post hoc test (*p* < 0.05) and ^ns^ indicates no significant difference. CON, conventional alkaline extraction; ENZ, enzyme-pretreated alkaline extraction; US, ultrasound-assisted alkaline extraction; ENZ+US, enzyme-pretreated and ultrasound-assisted alkaline extraction.

## Data Availability

The original contributions presented in this study are included in the article/Supplementary Material. Further inquiries can be directed to the corresponding author.

## Supplementary Materials

The following supporting information can be downloaded at: https://www.mdpi.com/article/10.3390/foods15183294/s1, Figure S1: Effects of ultrasonic amplitude on soluble N, Folin-Ciocalteu reducing capacity, DPPH radical scavenging activity, and ABTS radical scavenging activity (D) in enzyme-pretreated and ultrasound-assisted alkaline extract solution (ENZ+US) with 8 min of sonication; Figure S2: Eﬀects of ultrasonication time on soluble N, Folin-Ciocalteu reducing capacity, DPPH radical scavenging activity, and ABTS radical scavenging activity in enzyme-pretreated and ultrasound-assisted alkaline extract solution (ENZ+US) at 50% ultrasonic amplitude; Figure S3: Representative image of sodium dodecyl sulfate polyacrylamide gel electrophoresis (SDS-PAGE); Table S1: Chemical composition of mustard seed meal residue.
