# Supplementary material for: Effects of Extraction Methods on Physicochemical, Functional and Nutritional Properties of Protein Extracted from Mustard (Brassica juncea) Seed Meal Residue
Source: Foods. 2026 Sep 17;15(18):3294. doi: 10.3390/foods15183294 (PMC13606885; doi:10.3390/foods15183294)
Supplement: Supplementary file 1 [file foods-15-03294-s001.zip › foods-4496024-supplementary.pdf]

Supplementary material

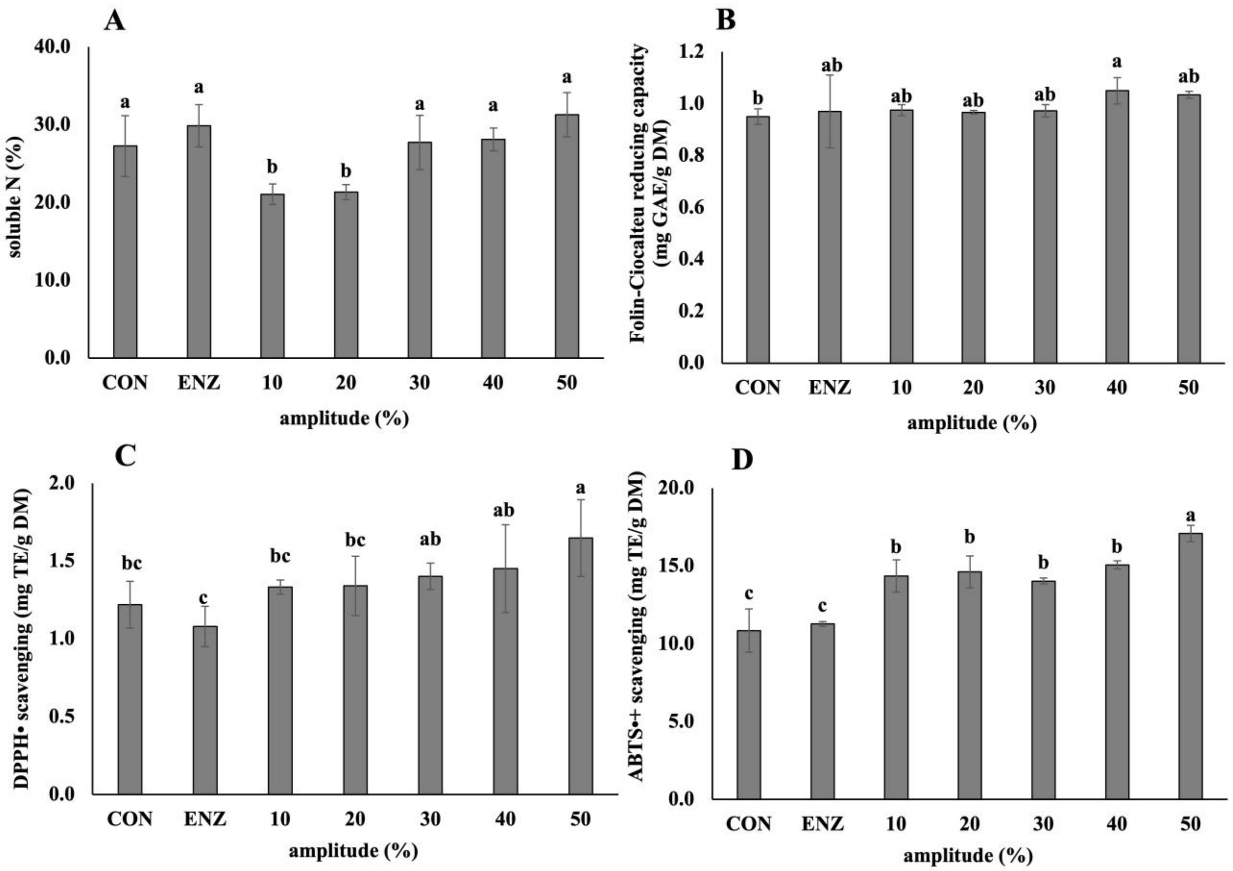

Fig. S1 Effects of ultrasonic amplitude on soluble N (A), Folin-Ciocalteu reducing capacity (B), DPPH radical scavenging activity (C), and ABTS radical scavenging activity (D) in enzyme-pretreated and ultrasound-assisted alkaline extract solution (ENZ+US) with 8 min of sonication. Values are expressed as mean ± SD. $^{a\text{-}c}$ letters demonstrate significant differences among samples (p < 0.05). Note: Soluble nitrogen in alkaline solution, calculated relative to that in the raw material, may comprise both storage protein nitrogen and non-protein nitrogen (e.g., isothiocyanates).

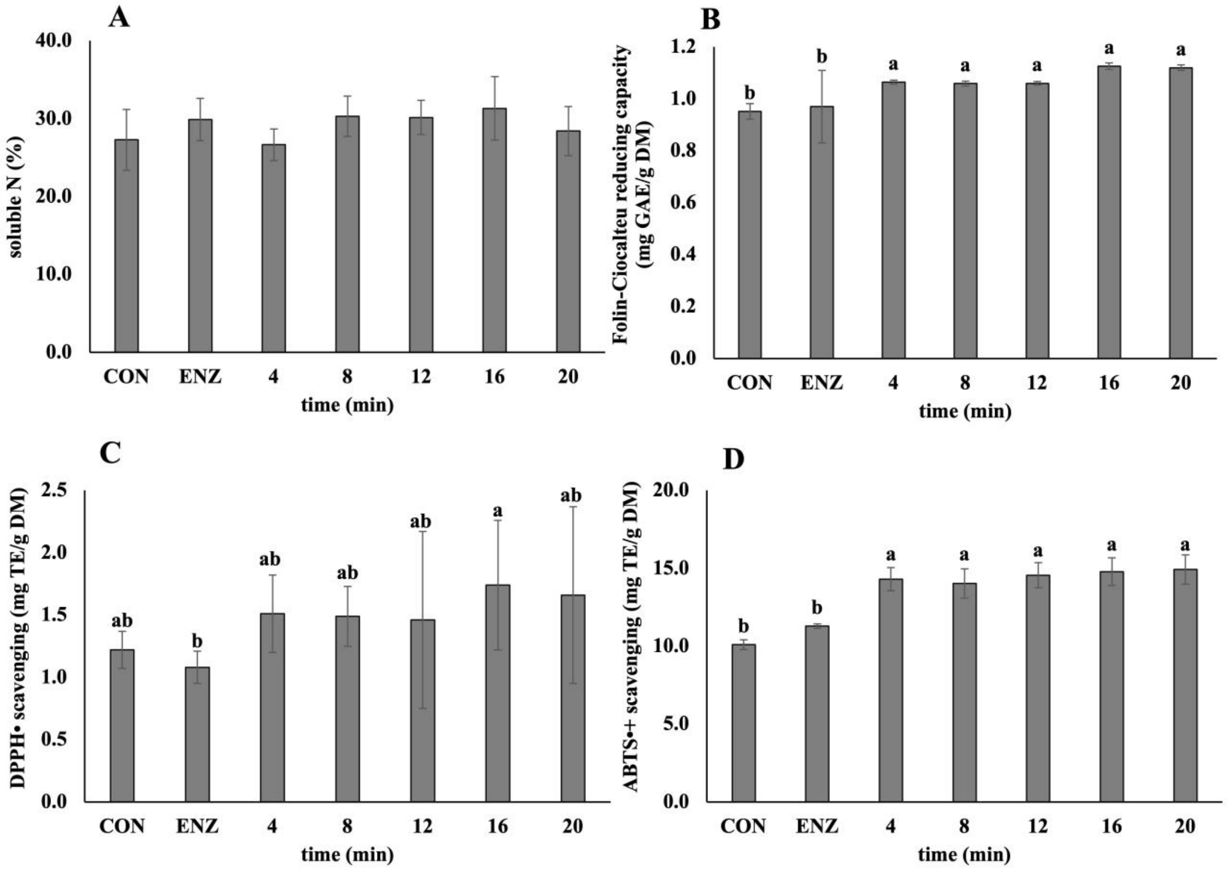

Fig. S2 Effects of ultrasonication time on soluble N (A), Folin-Ciocalteu reducing capacity (B), DPPH radical scavenging activity (C), and ABTS radical scavenging activity (D) in enzyme-pretreated and ultrasound-assisted alkaline extract solution (ENZ+US) at 50 % ultrasonic amplitude. Values are expressed as mean ± SD. $^{a\text{-}b}$ letters demonstrate significant differences among samples ($p < 0.05$). Note: Soluble nitrogen in alkaline solution, calculated relative to that in the raw material, may comprise both storage protein nitrogen and non-protein nitrogen (e.g., isothiocyanates).

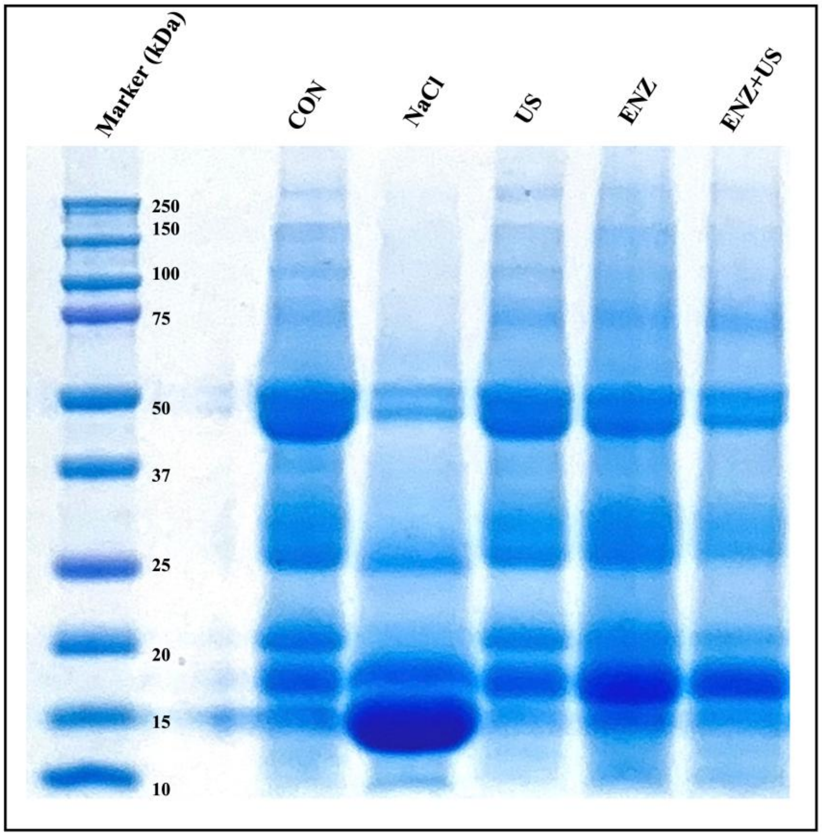

Fig. S3 Representative image of sodium dodecyl sulfate-polyacrylamide gel electrophoresis (SDS-PAGE). CON, Conventional alkaline extraction; ENZ, Enzyme-pretreated alkaline extraction; US, Ultra-sound-assisted alkaline extraction; ENZ+US, Enzyme-pretreated and ultrasound-assisted alkaline extraction; NaCl, Sodium chloride-assisted alkaline extraction.

Table S1 Chemical composition of mustard seed meal residue expressed as mean ± SD in g/100 g dried matter (DM)

Chemical composition	(g/100 g DM)
Protein	37.54 ± 0.17
Fat	18.74 ± 0.30
Moisture	4.16 ± 0.14
Ash	3.88 ± 0.17
Total carbohydrate	35.68 ± 0.40
Crude fiber	7.23 ± 0.17
